# An overview of motor skill performance and balance in hearing impaired children

**DOI:** 10.1186/1824-7288-37-33

**Published:** 2011-07-14

**Authors:** Venkadesan Rajendran, Finita Glory Roy

**Affiliations:** 1 Department of Rehabilitation Science, Holy Cross College affiliated to Bharathidasan University, Tiruchirappalli-620 002, India

## Abstract

Childhood hearing impairment is a common chronic condition that may have a major impact on acquisition of speech, social and physical development. Numerous literature states that injury to the vestibular organs may result in accompanying balance and motor development disorders. But still postural control and motor assessments are not a routine procedure in hearing impaired children. Hence, we aim to provide an overview on motor skill performance and balance in hearing impaired children.

## Introduction

Childhood hearing impairment is a significant public health problem, which is associated with long-term academic and communicative difficulties [1,2]. The prevalence of moderate to profound hearing loss in children, including sensorineural hearing loss and conductive hearing loss is 1 to 6 of 1000, of which, 10% have hearing levels that fall in the profound range [3-6]. Moreover, it is estimated that about 440 million children worldwide have hearing loss above 85 decibels, and this increases to about 800 million when the threshold is reduced to 50 dB [7,8]. Although many children have hearing impairments, each child is unique.

Newborn hearing screening has led to earlier identification and treatment of infants with hearing loss [7,8]. However, the earlier identification of childhood hearing impairment is considered critical for normal speech, language, cognitive and social development [4].

As routine screening does not include assessment of balance and motor deficits, physical therapy services are not included in the educational programme, unless obvious neurological or orthopedic disorders are diagnosed. However teachers and parents of these children often report inco-ordination, clumsiness and balance deficits which may hinder the child's optimal performance [9]. Moreover, many pediatric health care providers are often too busy or inadequately trained in conducting elaborate developmental screening tests during the regular clinics. These tests are performed only when the child present with an obvious deficit [10]. Hence, we aim to provide a concise description on balance and motor performance in hearing impaired children.

### Degree of hearing impairment

The degree of hearing loss explains the severity of hearing impairment. Table 1 shows the degrees of hearing loss according to American Speech-Language Hearing Association [11].

**Table 1:** Degree of hearing impairment

Degree of hearing loss	Hearing loss range (dB)
Normal	-10 to 15
Slight	16 to 25
Mild	26 to 40
Moderate	41 to 55
Moderately severe	56 to 70
Severe	71 to 90
Profound	91+

### Classification of hearing impairment

American speech-language hearing association has categorized hearing loss based on the part of the auditory system damaged. Accordingly there are three basic types of hearing loss: conductive hearing loss, sensorineural hearing loss, and mixed hearing loss [12].

#### Conductive hearing loss

Results from disruption in any of the mechanism, which conduct sound waves from external canal to the oval window. eg. Outer ear, ear drum or middle ear. It usually involves inability to hear faint sounds.

#### Sensorineural hearing loss (SNHL)

It is the most common type of permanent hearing impairment and results from damage to the inner ear or the nerve pathway from the inner ear to the brain.

#### Mixed hearing loss

It is a combination of both conductive and sensorineural hearing impairment.

Furthermore, hearing impairment may exist as unilateral or bilateral, and pre-lingual or post-lingual.

#### Unilateral

People have impairment in only one ear.

#### Bilateral

People have impairment in both the ears.

#### Pre-lingual deafness

It refers to hearing impairment that is sustained prior to the acquisition of language. eg. Congenital, early infancy.

#### Post-lingual deafness

It refers to hearing impairment that is sustained after the acquisition of language, which can occur as a result of disease, trauma or a side-effect of a medicine [13].

### Etiology

Davidson et al has proposed an etiologic classification which clarifies the interaction between time of insult, causation and time of expression of hearing loss. Most of the SNHL are idiopathic, and appears to occur almost twice as often as in developed countries. Where the cause is known it may be genetic; pre-natally acquired; peri-natally acquired; post-natally acquired; cranio-facial anomalies; and other [13,14].

#### Genetic

Almost 50% of permanent childhood hearing impairments have a genetic cause and influenced by consanguinity. It may be autosomal dominant, autosomal recessive and sporadic inheritance [13,15]. Parker et al studied the family history of hearing loss, the results pointing towards different genetic disorders with autosomal dominant, autosomal recessive and sporadic inheritance [16].

#### Prenatal Factors

Prenatal Infections such as rubella and toxoplasmosis are considered to be the main cause of prenatally acquired hearing impairment. Maternal exposure to alcohol, streptomycin, quinine and chloroquine phosphate may destroy neural elements of the inner ear and contribute to congenital hearing loss [13]. Sever et al has reported in his study that 38.7% mothers had antibodies to toxoplasmosis during pregnancy, and their children had double the risk of developing permanent childhood hearing loss by age 7 [17].

#### Perinatal factors

Perinatal factors such as prematurity, low birth weight, hypoxia, Apgar scores 0-4 at 1 min; and 0-6 at 5 min, ventilation required for five days or more, hyperbilirubinemia requiring transfusion, and admission to the neonatal intensive care unit for 48 hours or longer may predispose to permanent childhood hearing impairment [18]. Davis and wood found that one in 174 NICU graduates had a hearing impairment compared with one in 1278 non-NICU babies [19].

#### Post natal factors

There are many and varied postnatal causes for childhood deafness, which may result in sensorineural or conductive loss or both. The postnatal cause for childhood deafness includes bacterial meningitis, infection (eg. middle-ear infection, CMV infection), viral labyrinthitis (eg. measles and mumps), recurrent or persistent OME for at least 3 months, complications of otitis media, immunization, genetic causes, and head trauma with loss of consciousness or skull fracture [15].

## Hearing- a reflex process

Hearing is a reflex during the first couple of the month of life. Responses to sound at a threshold are yet to obtain during this period. The only response the child posses during this period is the startle reflex- a response to a loud sound, which is analogous to the protective reflex of hearing in animals. Following this, there is a development of comprehension hearing. The child begins to understand the sound with the help of experience and learning as the cerebral cortex takes over the control of auditory responses, with the simultaneous inhibition of the reflex hearing. Thus the child at the age of six to seven months does not give any response to a loud sound, but turn and look towards a faint sound that the child feels to be pleasant [20].

### Age appropriate hearing milestones

It is hard to know whether a child has a hearing problem or not. Hearing problems may be suspected if a child does not respond to sound or does not develop language skills appropriately. The National Institute on Deafness and Other Communication Disorders (NIDCD) has listed age-appropriate hearing milestones [21].

### Auditory development

Several hierarchies of auditory development have been proposed by numerous researchers and interventionists. Pollack et al has ranked auditory development in four stages [22].

#### Detection

Hearing a sound, pay attention to auditory signals, attend to sounds at a distance, search for and turn to the source of sound.

#### Discrimination

Discriminate the auditory stimuli, monitor the information and modify the speech production.

#### Identification

Select a word from group of words spoken, remember and recall information and language make cognitive judgements.

#### Comprehension

Synthesize meaning and respond to spoken language appropriately.

## Neurophysiology of postural control and motor development

It is necessary to understand the neurophysiology of postural control and motor development to get a clear construct of dysfunction. The ability to maintain a static posture (eg. sitting, standing) and dynamic posture (eg. walking) is operationally defined as static and dynamic balance respectively. Both the static and dynamic postural control are very important and necessary to execute movement [23,24]. The development and maintenance of postural control are known to be a pre-requisite for the performance of skilled movement. Simple or complex gross and fine motor tasks necessitate a person to maintain his or her center of gravity over the base of support. The development and maintenance of postural stability are a complex process that necessitates the involvement of multiple systems such as sensory system, central nervous system processing and co-ordination of motor output and the vestibular system. Three sensory systems (sensory triad of postural control) contribute to provide information from somatosensory, visual, and vestibular sources to maintain postural control, which is achieved by coordinated motor outputs. The visual and somatosensory system gathers information from the environment (eg. position in relative to other objects) and the vestibular system provides an internal reference (eg. head's orientation in space). Maturation of the vestibular system is responsible for the stabilization of eyes, head and body in space that helps to maintain an upright posture. The vestibular system is composed of two parts; (i) the vestibular ocular system, which maintains the visual stabilization, (ii) the vestibular-spinal system, which is responsible for the orientation of the body in space and maintenance of the postural tone, which is necessary for the development of motor milestone. It is normal for a human to have a certain amount of postural sway for various age groups and both the sexes have been documented. However, the child imitates the adult pattern of postural control by the age of seven to ten years. According to the sensory systems' perspective, young children depend on the visual system to maintain balance. As they grow older, there is a progressive domination of the somato-sensory system and the vestibular system [25-27].

## Patho-physiology of postural control and motor development in hearing impairment

Delayed postural development and motor development is a common sensorimotor impairment in profoundly deaf children. The vestibular end-organ and cochlea are closely related both anatomically and functionally. Therefore, injury or trauma prenatally, perinatally, or postnatally may cause damage to one or both systems [28-30]. Moreover, damage to portions of the vestibule-cochlear nerve is a presumed cause of sensorineural hearing loss, may include damage to both cochlear apparatus as well as the vestibular afferents [31]. The vestibular system is also critical for gaze stabilization. Thus damage to vestibular system causes gaze and balance impairments [32]. Potter and Silvermann has stated that many deaf children compensate for vestibular deficits through visual and kinesthetic systems to maintain static balance with eyes open or closed [33]. In a cross-sectional study Sharon et al studied 40 children with SNHL and found that 50% had abnormality in horizontal semicircular canal function, 38% had dysfunction in higher frequency canal function and 40% had abnormalities of saccular function [34]. Since damage to vestibular structures is known to cause the balance deficit, which may interfere with normal motor development, it has been postulated as the primary cause of motor deficit [35,36]. Crowe and Horak in a cross-sectional study found that hearing impaired children with sensory organization deficits have poor balance and motor proficiency in many areas [37]. Esther Hartman et al examined the motor performance in deaf elementary school children and found that deaf children had significantly more borderline and definite motor problems than the normative sample [38].

## Early identification and intervention

Early identification of childhood hearing impairment and prompt intervention are crucial for improved outcomes. Vestibular deficit related impairments and the efficiency of therapy intervention for such impairments in children are only recently documented [39-41]. In spite of the existing documentation, postural control and motor assessments are not a routine procedure in hearing impaired children. Moreover, in developing countries, early detection poses a significant practical challenge. Many pediatric health care providers are often too busy or inadequately trained to conduct an elaborate developmental screening test in regular clinics [42]. Consequently, balance and motor deficits in childhood are an overlooked entity and intervention to ameliorate these impairments is not provided. While testing the vestibular function, tests of canal and otolith function should also be included as semicircular canals and otoliths, mediate the vestibular ocular reflex and vestibule-spinal responses [32]. There is ample evidence that children with SNHL have concurrent motor and balance deficit. However, there is paucity in investigations of intervention for balance and motor performance deficits in hearing impaired children. Gheysen et al investigated the consequences of cochlear implantation on the motor abilities of deaf children and found that deaf children with cochlear implantation did not perform better on balance and motor skills than children without cochlear implantation [43]. Hence exercise intervention should be incorporated to improve balance and motor performance. Lewis et al found improvement in balance skill in 6-8 year old children following participation in balance and body awareness program [44]. Braswell and Rine found improvement in dynamic visual acuity, critical print size, and reading acuity following visual-vestibular exercises [45]. Rine et al reported improvement in sensory organization for postural control and halt of progressive motor delay following exercise intervention that focused on enhancement of sensory integrative postural control abilities [46].

## Summary

Childhood hearing impairments are a world-wide problem that causes the most serious limitation that can befall a child, as it prevents his optimal development. It has to be viewed as a multifaceted condition as a variety of factors determines the effect of hearing impairment on child's development. The focus of evaluation and treatment for these children is primarily on the language development. Therefore in order to minimize the adverse effects on normal development of hearing disorders, it is crucial to carryout screening examinations and appropriate interventions of balance and motor deficits, which enable early detection of these dysfunctions, which are often either not noticed or under estimated. It is also important to re-evaluate motor function in these children during the course of their childhood in order to assure early intervention.

## Competing interests

The authors declare that they have no competing interests.

## Authors' contributions

Both the authors contributed to the conception of the study and were involved in writing, revising and approving the final draft of the manuscript.
